# Natural Experiment Demonstrates That Bird Loss Leads to Cessation of Dispersal of Native Seeds from Intact to Degraded Forests

**DOI:** 10.1371/journal.pone.0065618

**Published:** 2013-05-31

**Authors:** Eleanor M. Caves, Summer B. Jennings, Janneke HilleRisLambers, Joshua J. Tewksbury, Haldre S. Rogers

**Affiliations:** 1 Biology Department, Pomona College, Claremont, California, United States of America; 2 Biology Department, Butte Community College, Oroville, California, United States of America; 3 Department of Biology, University of Washington, Seattle, Washington, United States of America; 4 Department of Ecology and Evolutionary Biology, Rice University, Houston, Texas, United States of America; 5 Department of Zoology, University of Cambridge, Cambridge, United Kingdom; 6 Department of Biology, Southern Oregon University, Ashland, Oregon, United States of America; University of Marburg, Germany

## Abstract

In healthy forests, vertebrate frugivores move seeds from intact to degraded forests, aiding in the passive regeneration of degraded forests. Yet vertebrate frugivores are declining around the world, and little is known about the impact of this loss on regeneration of degraded areas. Here, we use a unique natural experiment to assess how complete vertebrate frugivore loss affects native seed rain in degraded forest. All native vertebrate frugivores (which were primarily avian frugivores) have been functionally extirpated from the island of Guam by the invasive brown tree snake (*Boiga irregularis*), whereas the nearby island of Saipan has a relatively intact vertebrate frugivore community. We captured seed rain along transects extending from intact into degraded forest and compared the species richness, density and condition of the seed rain from native bird-dispersed tree species between the two islands. Considering seeds from native bird-dispersed species, approximately 1.66 seeds landed per 26 days in each square meter of degraded forest on Saipan, whereas zero seeds landed per 26 days per square meter in degraded forest on Guam. Additionally, on Saipan, 69% of native bird-dispersed seeds in intact forest and 77% of seeds in degraded forest lacked fleshy fruit pulp, suggesting ingestion by birds, compared to 0% of all seeds on Guam. Our results show an absence of seed rain in degraded forests on Guam, correlated with the absence of birds, whereas on Saipan, frugivorous birds regularly disperse seeds into degraded forests, providing a mechanism for re-colonization by native plants. These results suggest that loss of frugivores will slow regeneration of degraded forests on Guam.

## Introduction

Between one-third and one-half of Earth's land surface has been heavily influenced by humans [1], and 60% of tropical forestland (approximately 850 million hectares) is classified as secondary or degraded [2]. Degraded forests tend to harbour lower biological diversity, sequester less carbon, and differ in function and productivity when compared with nearby undisturbed forests [3]–[5]; however, specific restoration activities by humans in degraded forests can reverse these impacts and result in increased species diversity, ecosystem functioning, and carbon sequestration [5]–[7]. Some of this human-driven tropical forest restoration is achieved through active planting of native tree seedlings [8], but the majority of tropical forest regeneration takes place in a passive way, occurring via natural forest succession [9]. Passive regeneration is less resource and labor intensive than active restoration, but its success depends on a variety of factors, such as forest type, land-use history, and distance to nearest intact forest [10]. Thus, a key challenge for ecologists is to determine (1) the conditions under which passive regeneration can occur, and (2) which conservation measures most effectively assist passive regeneration [10].

Vertebrate frugivores assist in passive regeneration by transporting seeds to degraded areas from nearby intact forest [11]. Since approximately 90% of tropical forest tree species have fleshy fruits adapted for vertebrate dispersal [12], and vertebrate dispersers around the globe are under threat from overhunting, habitat fragmentation and invasive species [13], there is a critical need to understand the impact of frugivore loss on both the passive regeneration and active restoration of degraded forest.

Frugivores play an important role in the regeneration of degraded forests. Primates [14], [15], lizards [16], [17], bats [18], and birds [18]–[21] have all been shown to move seeds from primary to degraded habitat. Many frugivores travel long distances with seeds [22] and do not show an aversion to travelling through degraded forest areas [11], [23]; some bird species even show a preference for feeding, perching and roosting, and therefore defecating seeds, near gaps or edges [24]–[26]. In particular, vertebrate frugivores are important for moving the seeds of woody pioneer species and deep-forest, large-seeded species into degraded landscapes [27]–[31]. Several studies in regions with healthy frugivore communities have directly monitored seed rain and seedling recruitment in degraded areas adjacent to intact forests and have shown that vertebrate frugivores do move seeds, sometimes from far away, into degraded areas, often acting as the major (or only) source of native seed rain (e.g. [18], [19], [32]–[35]). The importance of vertebrate seed dispersal has been recognized by the implementation of restoration techniques which encourage dispersal, such as the planting of fruiting trees and ‘tree islands’ within degraded areas [36]–[38].

Despite the evidence that vertebrates play a role in regeneration of secondary forests, conservation or restoration of vertebrate frugivores is frequently ignored as a strategy for restoration of degraded lands (e.g. [39], [40]). The worldwide decline of vertebrate frugivore populations [41]–[43] may have negative impacts on forest regeneration [44]–[48], yet the magnitude of the effect of frugivore loss on seed dispersal to degraded forest is unknown. Here, we use a unique natural experiment to examine the impact of complete loss of vertebrate frugivores on seed dispersal from intact to degraded forests. Such natural experiments enable scientists to answer questions that cannot be addressed with a manipulative experiment for logistical or ethical reasons [49]; for example, removing all frugivores from large spatial areas is not feasible, thus a natural experiment provides a unique opportunity for understanding the magnitude of the seed dispersal provided by frugivores into degraded forest areas. While it is unlikely that forests around the world will lose all frugivores, examining the impact of such a dramatic change provides a critical view of the worst-case scenario.

The brown tree snake (*Boiga irregularis*) was accidentally introduced to the island of Guam in the late 1940s, causing a widespread loss of Guam's native forest bird species by the mid to late 1980s [50], [51]. Prior to the introduction of the brown tree snake, Guam's native forest avifauna consisted of 12 species, of which six were frugivorous. Predation by the brown tree snake led to the extirpation of five frugivorous species and the functional extirpation of the sixth [51]. Unlike many other places around the world that have lost native frugivores, Guam's forests have not been colonized by introduced avian frugivores that could play a similar ecological role to the extirpated species. The nearby island of Saipan provides a strong comparison to Guam, as it has similar forests, but in contrast to the silent forests of Guam, Saipan has no known snake population and healthy bird populations [52].

We investigated whether the species richness and density of bird-dispersed seeds varies between degraded forests on Saipan (with birds) and Guam (no birds). We also asked whether the proportion of seeds lacking fleshy fruit pulp (primarily due to handling by birds) differs between forests with birds (Saipan) and those without (Guam). Ours is the first study of which we are aware that utilizes a comparison between similar forests with and without avian frugivores to investigate the regeneration potential of degraded forests.

## Materials and Methods

### Ethics Statement

All field studies described here were conducted with the use of the necessary field permits. We obtained permission for the use of our study sites from the Government of Guam Forestry Division (for study sites on Guam), and from the Commonwealth of the Northern Mariana Islands Division of Fish and Wildlife (for study sites on Saipan). The field studies did not involve any protected or endangered species.

### Site Description

**Figure 1:**
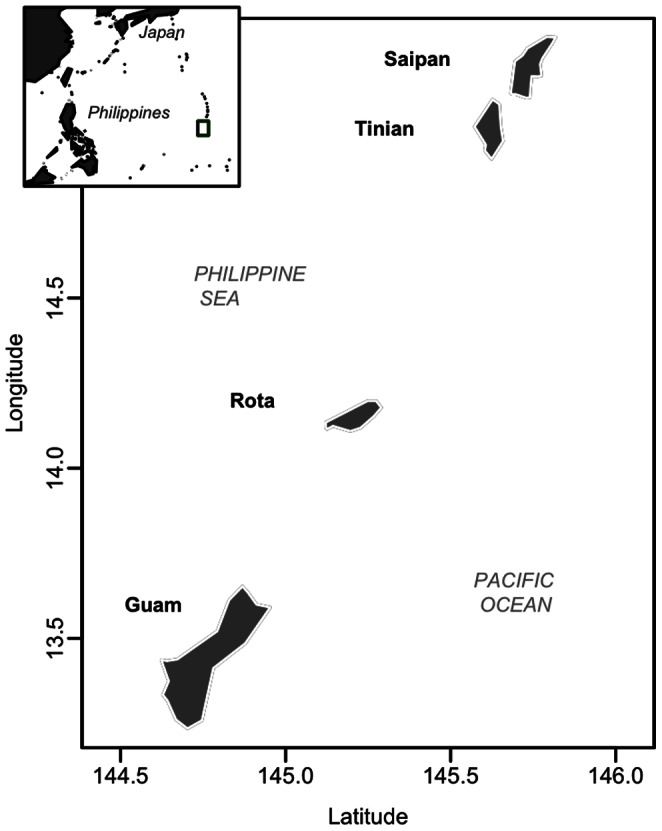
Map of the Mariana Islands. The locations of Guam and Saipan, the two islands used in this study, are shown.

This study was conducted on the Micronesian Islands of Guam (13°27′N, 144°46′E) and Saipan (15°11′N, 145°44′E). Both islands are at the southern end of the Mariana Island chain (Figure 1), and have climates with an average annual temperature around 27°C. The tropical climate of Guam averages 2586 mm of rainfall per year [53]. Saipan receives 1900–2300 mm of precipitation per year [54].

The primary forest type on Guam and Saipan is karst forest, which grows on a rugged karst limestone substrate; there are approximately 40 tree and shrub species in this forest type, but 8–12 species dominate. Large swaths of karst forest were destroyed on both Guam and Saipan in the 1930s and 40s as a result of World War II, and clearing has continued for development since then. When land is cleared, the rugged karst substrate is lost, and replaced by a topographically homogenous, red dirt substrate, which is typically colonized by the non-native leguminous tree, *Leucaena leucocephala* (Family: Fabaceae). Today, both islands have large areas of degraded forest composed of nearly monotypic stands of *L. leucocephala* with a sparse understory [55], [56]. Only 13% of Guam's remaining land area is intact karst forest, while 25% is degraded forest [55]. On Saipan, a smaller island with a proportionally larger impact of WWII, less than one percent of the total land area is intact karst forest, with 61% covered by degraded forest [56].

In this study, we define degraded forests as forests dominated by *L. leucocephala*. Degraded forest has a low (4–8 m in height) canopy that allows significant light to penetrate to the forest floor, whereas the intact karst forest is taller (7–13 m in height), creating a multi-storied, low-light environment. Narrow bands of transitional forest, characterized by a mixture of *L. leucocephala* and native forest trees, form the ecotone between degraded and intact forest.

### Seed dispersers

Birds and bats are the primary seed dispersers in the Mariana Islands. Prior to the introduction of the brown tree snake, six frugivorous birds dispersed seeds on Guam: the White-throated Ground-dove (*Gallicolumba xanthonura*), Mariana Fruit-dove (*Ptilinopus roseicapilla*), Mariana Crow (*Corvus kubaryi*), Guam Rail (*Gallirallus owstoni*), Bridled White-eye (*Zosterops conspicillatus*) and Micronesian Starling (*Aplonis opaca*) [51]. Only one frugivorous bird species, the Micronesian Starling, remains on the island of Guam, with a localized population likely numbering less than 500 individuals (J. Quitagua, L. Obra, D. Vice, pers.comm.). We have not seen Micronesian Starlings at any of the sites used for this study on Guam. Four of the six species formerly found on Guam are also native to Saipan (all except Mariana Crow and Guam Rail). In addition, the partially frugivorous Golden White-eye (*Cleptornis marchei*) is present on Saipan. Historically, the native Marianas Fruit Bat (*Pteropus mariannus*) was an important frugivore in forests on Guam and Saipan; however, populations of bats on both islands are functionally absent due to hunting and, on Guam, predation by the brown tree snake [57].

No non-native species have taken over the entire functional role of the native seed dispersers on Guam, although a small number of tree species may be dispersed by invasive mammals. One non-native avian frugivore, the Philippine Turtle-dove (*Streptopelia bitorquata*), is found on both Guam and Saipan, typically along roadsides; it is rarely seen in intact karst forest on Guam, and thus is unlikely to be playing a significant role in seed dispersal. Introduced rats, primarily *Rattus diardii* (sensu [58]), are present in forests on both Guam and Saipan [59], and introduced pigs (*Sus scrofa*) and Philippine deer (*Rusa marianna*) are present on Guam. While all three species consume fruit and seeds, their role in seed dispersal is unclear. In other locations, invasive rat species such as *Rattus diardii* and *Rattus rattus* are primarily considered seed predators [60], [61] and rat eradication projects have resulted in increased seedling recruitment [62]. However, invasive rats occasionally disperse seeds [63] and may be effective dispersers for very small seeds [64]; their role in the Marianas is unstudied. Germination experiments from scat samples of deer and pigs on Guam show that deer are not effective dispersers, while pigs disperse seeds from at least three tree species, *Morinda citrifolia* (native), *Ficus prolixa* (native), and *Carica papaya* (naturalized non-native), as well as several non-native herbaceous weed species (A. Gawel, unpub. data). In this study, we capture seed rain exclusively from flying frugivores in order to quantify the effect of native frugivore loss on seed rain into degraded forests, and thus, we do not measure dispersal by feral pigs or rats.

### Site selection

We selected three sites on each island where degraded forest bordered intact karst forest. Transitional zones between the intact and degraded forests were no more than 10 m wide at any site. On Guam, the three sites were located on the northern half of the island, where intact karst forest is prevalent along cliff lines. On Saipan, we selected a northern, a central and a southern site, also where intact forest remains along cliff lines. Sites were separated from each other by a minimum of 5 km.

### Bird-dispersed tree species

Our study focused on seeds from native, bird-dispersed tree species. We designated a tree species as ‘bird-dispersed’ if its seed is covered by fleshy fruit pulp, birds have been observed consuming either the flesh or the entire fruit, and each seed is small enough to be consumed or carried by the largest frugivorous bird species that once occurred on the island. Additionally, we have found seeds from two common species, *Premna obtusifolia* and *Psychotria mariana*, in bird scat, providing additional support for their status as ‘bird-dispersed’. Table 1 includes a summary of the bird species seen consuming fruit from each tree species. All recorded observations of avian frugivory in the Marianas come from observations of birds rather than trees, thus we do not have any quantitative estimate of the proportion of fruit crops consumed by birds.

**Table 1:** Presence and dispersers of bird-dispersed tree species and their seeds on Guam and Saipan.

Family	Species	Bird species seen consuming fruit^1^	Guam			Saipan		
			Site 1	Site 2	Site 3	Site 1	Site 2	Site 3
**Euphorbiaceae**	*Melanolepis multiglandulosa*	BRWE, WTGD, GOWE, MFD, MIST	Traps			Both	*	Both
**Verbenaceae**	*Premna obtusifolia*	WTGD, BRWE, GOWE, MFD, MACR, MIST	Both	Both, Fruit	Both, Fruit	Both	Traps	Both
**Rubiaceae**	*Psychotria mariana*	GOWE^2^	Traps	Traps		Both	Traps	Traps

Disperser identity is based on bird observations reported in Craig [95] and Jenkins [96]. No tree species were present in intact karst forest survey transects but not in seed trap contents. A tree species that was present in both intact karst forest survey and in seed trap contents is indicated below by ‘Both’. ‘Traps’ indicates a tree species was present in seed trap contents, but was not present in intact karst forest surveys. ‘Fruit’ indicates that the species was seen fruiting during the forest surveys; if a species was found in seed traps (‘Both’ or ‘Traps’), we also assume that it fruited during the study.

1 MFD = Mariana Fruit-dove, WTGD = White-throated Ground-dove, BRWE =  Bridled White-eye, GOWE = Golden White-eye, MACR = Mariana Crow, MIST = Micronesian Starling. We lack information on fruit in the diet of the Guam Rail.

2 Other bird species likely disperse *Psychotria mariana*, but systematic observations of fruiting trees have not been conducted.

### Seed traps

At each site, we set up seed traps in intact karst forest and along three parallel transects from the intact forest/degraded forest boundary into degraded forest. Circular seed traps (0.5 m^2^) were constructed using polyvinyl hoops with screen door netting added to make a basket, and suspended from trees at a height of 1.3 m. By hanging the traps, we measured only dispersal by volant frugivores, and not by terrestrial frugivores. Beginning with a trap in the transitional forest, three parallel transects were established a minimum of 10 m apart from one another, and a trap was placed every 10 m along each transect into degraded forest out to a distance of 100 m. To sample the intact forest seed rain, four additional seed traps were set up in the adjacent intact karst forest 10 m from the start of each transect. These four traps were arranged in a 5-m by 5-m square, with a trap at each corner; we placed traps in this arrangement because a small number of transects contained a small cliff or other boundary within the native forest which prevented us from creating 40-meter straight line seed trap arrays. The square array of seed traps at the base of each transect ensured all native forest areas were sampled using the same method. Since the forest canopy is low, seed traps spaced 5 meters apart are unlikely to be underneath the same tree canopy and thus can be considered independent samples. We considered seed traps to be ‘intact forest traps’ if they were within intact forest or in the transitional zone because native seeds could fall into any of these traps without the help of birds. Traps were designated as ‘degraded forest traps’ if they were located in degraded forest (*i.e.* traps at 10–100 m from the intact/degraded forest boundary).

After 26 days, seed trap contents were collected and dried in a drying oven to aid in leaf litter removal. All seeds from bird-dispersed tree species were counted and divided into “whole fruit” and “de-pulped” categories based on the presence or absence of a fleshy fruit covering.

### Forest comparisons

Since this was a large-scale comparative study between two islands, and our main comparison is between degraded forests on Guam and Saipan, we could not control for island-specific differences in species richness of the forests [65], [66]. However, we took several steps to ensure that differences in degraded forest seed rain truly reflected differences in movement from intact to degraded forest, not differences in intact karst forest or degraded forest diversity. As described above, we sampled seed rain in intact karst forest in addition to degraded forest because intact forest is the most likely source of seeds in degraded forest traps. We qualitatively compared intact karst forest composition between Guam and Saipan using three parallel straight-line 25-m transects, separated by more than 5 m, in intact karst forest at each site. On each transect, we recorded the presence or absence of bird-dispersed tree species (Table 1, Table S1). The degraded forest at all sites on both islands was dominated by *L. leucocephala*, however a few sites contained remnant trees of native bird-dispersed species as well. We recorded the location of remnant trees in degraded forest and took them into account in our analyses, as our objective was to assess movement of seeds from intact karst forest, not dispersal within degraded forest.

### Analyses

We investigated whether forest type (intact vs. degraded) and island (Guam vs. Saipan) affected the species richness and density of native bird-dispersed seeds, and the proportion of native de-pulped seeds. We only used data on native bird-dispersed tree species in our analyses, to evaluate the role of birds in the regeneration of native tree species in degraded forest. Thus, we excluded data from two non-native, bird-dispersed naturalized species, *Carica papaya* and *Triphasia trifolia*, and one non-native, wind-dispersed invasive species, *L. leucocephala*, despite the presence of seeds in the traps. We did not use distance from the intact forest edge as a continuous explanatory variable for the analyses because preliminary analyses indicated that response variables were not a simple linear (or log-linear) function of distance (Figure S1). The sampling unit for the analyses was the individual trap, blocked by site and forest type.

To account for the few remnant native trees in degraded forest, we excluded seeds from traps in degraded forest within 10 m of a fruiting remnant native tree. The only native species we found fruiting in the degraded forest was *Premna obtusifolia*, and only eight of the 180 degraded forest traps (4%) were located within 10 m of these fruiting adults. Five of these eight traps were located on Guam, where their contribution would have increased the density and richness of native bird-dispersed seeds in degraded forest, making their omission a conservative approach. Results were qualitatively similar even when including these traps.

To determine whether the species richness and density of native bird-dispersed seeds found in degraded forest seed traps was greater on Saipan than Guam, we used generalized linear mixed effects models with a Poisson error distribution [67]. We used the number of species per trap as the response, and island, forest type, and an island by forest type interaction as fixed effects and site as a random effect. We also tried a binomial error distribution for the species richness analysis, with the proportion of native bird-dispersed species observed in each trap out of the total number of bird-dispersed species found in all traps as the response. This analysis yielded qualitatively similar results; however, we chose not to use this model structure because we do not with certainty know the actual number of native bird-dispersed species present in our sites. Though we performed transect surveys of our study sites, there were bird-dispersed species in our study areas that were not fruiting at the time of the experiment, and thus did not appear in our seed traps (Table S1).

For each analysis, we fit five models; (1) null (intercept only); (2) island effects only; (3) forest type effects only; (4) island and forest type effects; and (5) island, forest type, and their interaction. We identified the best fitting model with Akaike's Information Criterion (AIC) values. We chose the simplest model (*i.e.* fewest explanatory variables) within two AIC units of the best-fitting model [68]. If birds play an important role in dispersing native tree seeds to degraded forests, the full model with an interaction between island and forest type will best fit observed data, with the interaction coefficient showing that the difference in the species richness or density of native seeds in degraded versus intact karst forest is smaller on Saipan than on Guam.

We conducted additional analyses to test whether seed species richness or density is significantly greater in degraded forests on Saipan than on Guam, as hypothesized. We fit a simpler model using degraded forest data only, and tested for island effects using a similar generalized linear model structure. We then selected the best fitting model using AIC values, as described above. The best-fit model for seed density showed overdispersion, which we corrected for by adding an observation-level random effect [69].

We used two separate analyses to determine whether the proportion of bird-dispersed seeds with the pulp removed (likely by birds) differed between the two islands, and between intact and degraded forest on Saipan. We used generalized mixed effects models for both analyses, specifying a binomial error distribution and site as a random effect. We used AIC values to identify best fitting models. We expected to find a higher proportion of seeds with pulp removed on Saipan than on Guam (*i.e.* best-fitting models include island), and a higher proportion of seeds with pulp removed in degraded than in intact karst forest on Saipan.

All statistical analyses were performed using R version 2.13.0 [70] with the lme4 package (published March 8, 2011).

## Results

The species composition of the intact forests on Guam and Saipan were qualitatively similar as shown by transect surveys (Table 1). Similarly, seed rain in intact forest on both islands contained seeds from three native, bird-dispersed tree species: *Melanolepis multiglandulosa, Premna obtusifolia*, and *Psychotria mariana* (Table 1). In addition, nearly all traps on both islands contained seeds from the non-native species *Leucaena leucocephala*, although the seed rain in degraded forest differed between islands (summarized in Table 2). All seeds from native, bird-dispersed tree species found in traps in degraded forest on Saipan were from three species: *Melanolepis multiglandulosa, Premna obtusifolia*, and *Psychotria mariana*.

**Table 2:** Summary of seed trap contents on each island, and in each forest type.

	Forest type	Guam	Saipan
**# (%) of traps with seeds from native bird-dispersed tree species**	Intact	17 (37.8%)	32 (71.1%)
	Degraded	5^1^ (5.6%)	41^2^ (45.6%)
**# (%) of traps with ** ***Leucaena***	Intact	26 (57.8%)	22 (48.9%)
	Degraded	88 (97.8%)	83 (92.2%)

In total, we had 45 traps on each island in intact forest, and 90 traps per island in degraded forest.

1 All five of these traps were under either native or naturalized remnant fruiting trees and contained whole fruits covered with fleshy fruit pulp.

2 Three of these traps were under native or naturalized remnant fruiting trees.

**Figure 2:**
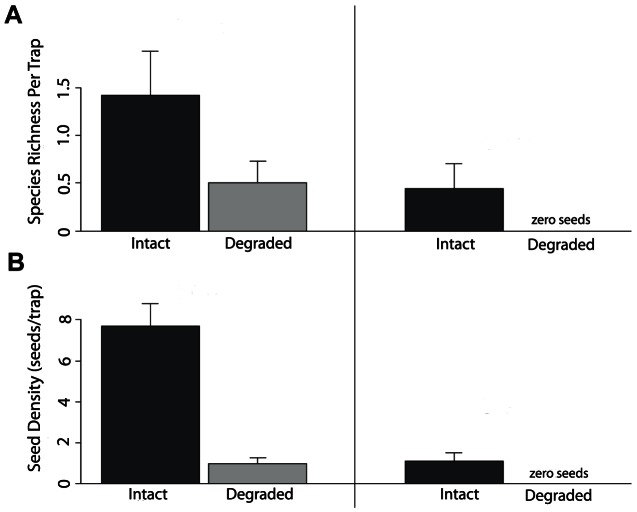
Mean (A) species richness and (B) density of native bird-dispersed seeds on Guam and Saipan. Dark grey bars indicate intact karst forest, while light grey bars indicate degraded forest. Error bars represent standard error. Means were calculated using raw data on native bird-dispersed seeds per trap (0.5 m^2^); in degraded forests, we used only seeds found in traps with no remnant native trees nearby.

After excluding seeds captured in traps located underneath remnant native trees in degraded forest on each island, significantly more native, bird-dispersed species were captured per square meter of degraded forest on Saipan than on Guam, where none were caught. Based on coefficients from the best-fit statistical model, which incorporates the random effects of transects within sites, 1.66 seeds of native bird-dispersed tree species fall every 26 days in each square meter of degraded forest on Saipan. The species richness and density of seeds from native bird-dispersed tree species are best explained by models including island, forest type and their interaction (Table 3). Native seed species richness was greater on Saipan than on Guam, and greater in intact than in degraded forests (Figure 2A). The density of seeds from native bird-dispersed trees was higher on Saipan than on Guam and lower in degraded forests than intact karst forests (Figure 2B).

**Table 3:** Comparison of statistical models containing different combinations of Island and Forest type as explanatory variables for the richness of (column 1) and density (column 2) of seeds that come from native bird-dispersed species.

Model	Native richness		Seed density	
	AIC Values	Δ AIC	AIC Values	Δ AIC
**Full model**	**176.47**		**865.46**	
**Island, Forest type**	192.69	16.22	872.31	6.85
**Forest type**	207.05	30.58	883.03	17.57
**Island**	243.56	67.09	1349.03	483.57
**Null (intercept)**	257.92	81.45	1359.75	494.29

AIC values of best fitting models are in bold.

The proportion of seeds from bird-dispersed tree species that were de-pulped was lower on Guam than on Saipan. On Guam, zero seeds were de-pulped; on Saipan, 68.5% of seeds in intact forest, and 76.3% of seeds in degraded forest, were de-pulped. Because there were no native bird-dispersed seeds away from remnant trees and no de-pulped seeds in degraded forest traps on Guam, we were unable to fit a full model with island, forest type, and their interaction to these data. Instead, we compared the proportion of seeds that were de-pulped out of all seeds found on each island. The model containing island as a predictor fit better than the model without island, indicating that proportion of seeds with pulp removed differs greatly between Guam and Saipan (delta AIC  = 14.47). The proportion of bird-dispersed seeds with pulp removed was slightly higher in degraded forest than intact karst forest on Saipan (0.77 vs. 0.69), but the AIC value for the model including forest type as an explanatory variable was within 1.5 AIC units of the null model (including only an intercept), indicating that forest type does not significantly improve model fit.

## Discussion

Avian frugivores act as critical links between intact karst forest and degraded forest in the Mariana Islands. We found increased species richness and density in the seed rain landing in degraded forest on Saipan compared with Guam, likely due to the absence of frugivorous birds in the forests on Guam. Zero seeds/m^2^ from bird-dispersed tree species were dispersed into degraded forests on Guam, indicating a loss of seed dispersal services on this island. In contrast, approximately 1.66 native bird-dispersed seeds/m^2^ are dispersed into degraded forest on Saipan per 26 days. While this is a small number of seeds, these seeds provide the potential for regeneration of native trees within the degraded forest. There are few wind-dispersed tree species in the Marianas, and Mariana Fruit Bats are functionally extinct on Guam and Saipan, leaving birds and gravity as the primary modes of dispersal. This is the second study to document the loss of an ecosystem function once provided by birds on Guam; the previous demonstration focused on birds as pollinators [71].

Vertebrate dispersers play a key role in catalyzing regeneration of native forest. In systems with intact frugivore communities, vertebrate dispersal from native to degraded forest is evident from the abundance of seeds and seedlings from fleshy-fruited species found in degraded forest [20], [29], [33], [72]. A study of forests on the island of Saipan suggests that vertebrate dispersal is important for bringing native seeds to degraded forest because most of the native seeds and seedlings sampled in degraded forest had small, fleshy fruits typically dispersed by birds [73]. In our study, the native species dispersed into seed traps in degraded forest on Saipan were, in descending order of abundance, *Premna obtusifolia*, *Psychotria mariana*, and *Melanolepis multiglandulosa*. These species produce large fruit crops, and birds have been observed eating all three species. *M. multiglandulosa* is a pioneer species in this system, as it is only found in gaps and edges of forests.

Initial dispersal events from intact to degraded forests are important because they can initiate the regeneration of degraded areas. Once a native fleshy-fruited tree becomes established in degraded forest, it provides habitat, food, and perching and roosting sites for frugivores, which in turn will bring more seeds from the nearby intact forest [28], [36], [74], [75]. Without birds bringing in early pioneer species, we predict that regeneration of native species in degraded forest will progress more slowly on Guam than on Saipan.

While the focus of this study is on seed dispersal into degraded forests, we also found significant differences in seed rain in intact karst forests (Figure 2). Seed traps placed in intact forest on Guam collected a lower richness and density of seeds from native bird-dispersed species than on Saipan. Using transect surveys (Table 1), we showed that the forests on Guam and Saipan were qualitatively similar in species composition; thus, we believe that these differences in abundance and diversity reflect the lack of dispersal by birds in Guam's forests, rather than differences in species composition or inadequate sampling. Frugivorous birds often consume seeds from one tree, then perch on another and defecate, leading to a higher richness of seeds within a single seed trap. On Guam, the only seeds found in forest traps likely originated from tree species with canopies overhanging the traps since no volant dispersal agents are present; this potentially explains the lower richness and density of seeds in traps placed in Guam's intact karst forests compared to traps on Saipan (Figure 2).

In addition to moving seeds into degraded forests, birds may influence the likelihood that seeds germinate. When birds ingest fruit, they remove the fleshy fruit pulp and scarify the seed, processes that enhance germination in some species, but do not affect or even reduce seed germination in others [76]–[81]. A recent study of 15 New Zealand tree species found a statistically significant but biologically unimportant effect of birds on the proportion of seeds that germinated, suggesting that germination of most tree species would be unaffected by the loss of ingestion by birds [82]. In our study, none of the native bird-dispersed seeds found on Guam had their fleshy fruit removed, as was expected since birds are absent. On Saipan, however, nearly two-thirds of all bird-dispersed native seeds found in traps lacked fruit pulp. In a separate study, germination experiments in an outdoor nursery for two of the common bird-dispersed species, *Premna obtusifolia* and *Psychotria mariana* showed increased germination of handled seeds over whole fruit (Rogers, unpub.). Thus, the loss of birds on Guam may affect germination due to the loss of seed handling for at least these two species, regardless of whether the seeds land in degraded or intact karst forest. However, this positive effect of bird handling may not be a general phenomenon in Mariana plants, and thus requires additional study. Un-dispersed seeds may also experience lower levels of germination due to increased natural enemy attack near the parent trees (i.e. Janzen-Connell effects) [83].

There are at least two aspects of the present study that limit our capacity to predict the full impact of birds on degraded forest regeneration. First, we did not assess rates of germination from the seed bank in degraded forest. It is possible that native species were at one point deposited in the seed bank by birds, but are waiting for the right conditions to germinate [84]. However, it is unlikely that native seeds would still be viable if they fell prior to land clearing (10–80 years ago) or were brought to the degraded forests when bird dispersers were present on the island (>25 years ago), since many tropical seeds are viable for short periods of time [27], [85]. Second, we did not assess rates of germination and survival of seeds and seedlings in degraded forest, so it is possible that bird dispersal of native seeds into degraded forest does not lead to regeneration of those species. There are several lines of indirect evidence that suggest that at least some native seeds, once dispersed into degraded forests, can germinate and survive. On Saipan, native seedlings have been observed growing in the understory of many *L. leucocephala*-dominated forests [73], [86]. In addition, we have observed forests on Saipan that have a soil substrate similar to that indicative of cleared forest, but that support a forest cover similar to intact forest with only a few large adult *L*. *leucocephala* trees. These areas were likely bulldozed many years ago changing the substrate from karst to soil, then were initially colonized by *L. leucocephala*, which has since been gradually replaced by native forest species, resulting in a forest with a similar composition and diversity to intact karst forest. Finally, *L. leucocephala* does not appear to invade intact karst forest successfully, perhaps because it does not compete well with native trees in undisturbed forest [86]. If *L. leucocephala* is indeed a poor competitor with native trees, then karst forest regeneration in areas currently dominated by *L. leucocephala* may be successful without labor-intensive management of *L. leucocephala* once native seedlings get established.

Bird populations worldwide are in decline due to habitat loss, climate change, introduced disease, and invasive species [41], [47]. Nearly one-quarter of all frugivorous birds are prone to extinction [42], and the decline will impact the ecosystem services they provide [22], [47], [87]–[89]. Birds provide an important ecosystem service when they move seeds from native to degraded forest. In the Marianas, the intact karst forest harbours greater tree species diversity than degraded forest, including many species of cultural importance (*e.g. Intsia bijuga, Premna obtusifolia, Eugenia* spp.), and provides habitat for wildlife (*e.g.* coconut crabs, Mariana Fruit Bats, Micronesian Starlings). In addition, intact forests are highly productive, and often store more carbon than degraded forests due to the abundance of large, old-growth trees as opposed to small, successional trees found in degraded forests, as well as a higher canopy with understory, mid-story and canopy tree levels [6], [7], [90]; we suspect this is also the case in the Marianas. Thus, if seed dispersal by birds does speed up regeneration of degraded forest, frugivorous birds deserve recognition for providing a key ecosystem service for the people of the Mariana Islands.

Land managers should explicitly consider the role of seed dispersers in degraded forest regeneration. In locations where frugivore populations are healthy, conservation of intact forest and frugivore communities will ensure that passive regeneration of degraded forest is not limited by dispersal. In locations where frugivore communities are reduced or absent, conservation of intact forest, restoration of frugivore communities, and intensive management may all be needed to restore or replace the ecosystem services provided by frugivores. Conservation of remnant intact forest patches should be prioritized because these remnants serve as a seed source for degraded forests; forest regeneration is most likely to occur in areas where both older remnant forest stands and seed dispersers—such as avian frugivores—persist nearby [91], [92]. Restoration of frugivore communities should also restore the ecological functions they provide (e.g. seed dispersal). In cases where frugivores are absent or reduced in abundance and their restoration is impossible, management efforts will need to include active planting of seeds and seedlings in degraded forest. There has been little effort to restore native karst forest speces to areas currently covered with degraded forest in the Marianas, except for a few small-scale plantings by volunteers across the islands. Our results suggest that these efforts are necessary on Guam because birds no longer provide seed dispersal to degraded forests. In addition, managers should consider re-introducing frugivorous birds within snake exclosures in order to restore seed dispersal services. Reintroduction of native frugivores should be a top priority restoration strategy, since non-native frugivores may not replace the ecological functions of native frugivores [93]. Finally, in situations where populations of native frugivores cannot be conserved, introduction of analogue frugivores should be considered, in order to at least partially maintain the ecosystem services they provide [94]. On Guam, this could mean introducing analogue disperser species that are able to co-exist with brown tree snakes.

The island of Guam provides the worst-case scenario for the impact of frugivore loss on seed rain in degraded forest, and shows a stark view of the disruption of ecosystem services provided by frugivores, evident here as the complete cessation of seed rain into degraded forests. The unequivocal absence of adequate frugivore populations on Guam both demands immediate action, and provides an opportunity for managers to test and advance new methods for addressing seed dispersal disruptions.

## Supporting Information

Figure S1 **Seed rain in degraded forest with respect to distance from intact karst forest boundary.**
(TIFF)

Table S1 **Summary of tree species found in forest surveys or seed traps, with biogeographic status.**
(DOC)
