# Supplementary figures and images for: Natural Experiment Demonstrates That Bird Loss Leads to Cessation of Dispersal of Native Seeds from Intact to Degraded Forests
Source: PLoS One. 2013 May 31;8(5):e65618. doi: 10.1371/journal.pone.0065618 (PMC3669269; doi:10.1371/journal.pone.0065618)

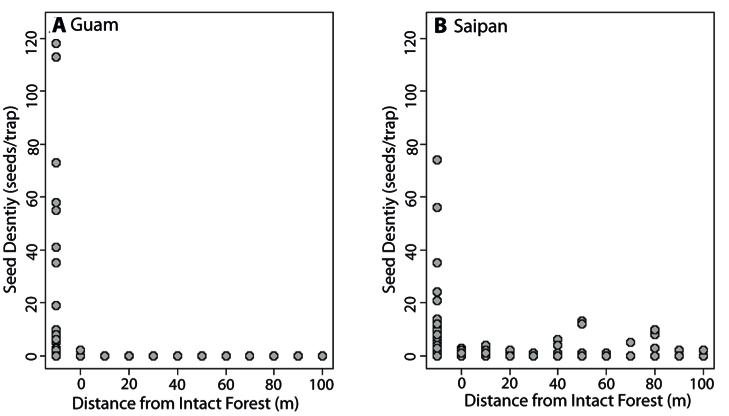

Supplement: Figure S1 — Seed rain in degraded forest with respect to distance from intact karst forest boundary. (TIFF) [file pone.0065618.s001.tiff]
