# Supplementary material for: Natural Experiment Demonstrates That Bird Loss Leads to Cessation of Dispersal of Native Seeds from Intact to Degraded Forests
Source: PLoS One. 2013 May 31;8(5):e65618. doi: 10.1371/journal.pone.0065618 (PMC3669269; doi:10.1371/journal.pone.0065618)
Supplement: Table S1 — Summary of tree species found in forest surveys or seed traps, with biogeographic status. (DOC) [file pone.0065618.s002.doc]

**Supporting Information**

**Table S1. Summary of potentially bird-dispersed tree species found in forest surveys or seed traps.** ‘G’ indicates the species was found on Guam. ‘S’ indicates the species was found on Saipan. * indicates that the species was seen fruiting during transect surveys. The presence of seeds in the seed traps also indicates that the species was fruiting.

| **Species** | **Biogeographic Status** | **Forest survey** | **Seed trap** |
| --- | --- | --- | --- |
| *Aglaia mariannensis* | native | G* | No |
| *Aidia cochinchinensis* | native | G, S | No |
| *Carica papaya* | non-native, naturalized | G, S* | S |
| *Eugenia reinwardtiana* or *palumbis* | native | G*, S | No |
| *Guamia mariannae* | native | G, S* | No |
| *Maytenus thompsonii* | native | G | No |
| *Melanolepis multiglandulosa* | native | S | G, S |
| *Morinda citrifolia* | native | G* | No |
| *Premna obtusifolia* | native | G*, S | G, S |
| *Psychotria mariana* | native | S | G, S |
| *Triphasia trifolia* | non-native, naturalized | G* | G |
